# Long‐term oncological outcomes of local excision versus radical resection for early colorectal cancer in young patients without preoperative chemoradiotherapy: a population‐based propensity matching study

**DOI:** 10.1002/cam4.1508

**Published:** 2018-05-03

**Authors:** Bin Cao, Li Min, Shengtao Zhu, Haiyun Shi, Shutian Zhang

**Affiliations:** ^1^ Beijing Key Laboratory for Precancerous Lesion of Digestive Diseases Department of Gastroenterology Beijing Friendship Hospital Capital Medical University National Clinical Research Center for Digestive Diseases Beijing Digestive Disease Center NO. 95, Yong'an Road Xicheng District Beijing China

**Keywords:** Colorectal cancer, local excision, oncological outcomes, radical resection, young patients

## Abstract

The incidence and mortality of colorectal carcinoma are rising in young adults. This population‐based propensity matching study aimed to compare long‐term oncological outcomes of local excision with radical resection for early localized colorectal cancer (CRC) in young patients without preoperative chemoradiotherapy. Patients under 45 years old with T1 colon or rectal adenocarcinoma who underwent local excision or radical resection were included from the Surveillance, Epidemiology, and End Results (SEER) database between 1998 and 2014. Survival curves were plotted using the Kaplan–Meier method. Cancer‐specific survival (CSS) was compared using adjusted hazard ratios (HRs) between local excision and radical resection. After propensity score matching procedure, total of 1719 patients were included in the analysis, among which 573 treated with local excision and 1146 treated with radical resection. The median follow‐up was 80 months (interquartile range(IQR): 37–132), with 1074 patients followed for ≥5 years and 508 patients followed for ≥10 years. Five‐year CSS of local excision versus radical resection was 93.4% versus 96.7% for colon cancer and 96.6% versus 98.4% for rectal cancer. Ten‐year CSS of local excision versus radical resection was 91.4% versus 94.0% for colon cancer and 92.8% versus 96.7% for rectal cancer. On multivariable analysis, compared with radical resection, local excision was not associated with inferior CSS for colon (HR 1.74, 95% CI: 0.92–3.29, *P *= 0.090) and rectal cancer (HR 2.16, 95% CI: 0.99–4.71, *P* = 0.052). There is no evidence of differential long‐term oncological outcomes between local excision and radical resection. These findings supported clinical application of local excision for early colon and rectal cancer in young adults.

## Introduction

CRC is the third most common cancer and the third leading cause of cancer‐related deaths in developed countries 1. The improvement and availability of colon screening make it possible to distinguish colorectal carcinoma in an early stage and contribute to declining incidence and mortality rates of CRC 2. However, incidence and mortality in young patients are increasing 3, 4, 5. It is reported that CRC‐related mortality increased by 13% in those aged <50 years in last decades 6.

Radical surgery combined with lymphadenectomy is still considered to be the gold treatment for CRC. Adverse events after colorectal radical resection range from 20% to 40%, including anastomotic leakage, urinary dysfunction, and/or poor functional outcomes 7, 8. Local excision for early CRC has been accepted as an alternative surgical option, with a reduced risk of mortality and a better quality of life compared with radical resection 9. Differ from colostomy after radical resection, for instance, local excision for low rectal cancers benefit in sphincter preservation 10. An ideal surgical approach for young patients should balance efficacy of oncologic control and quality of life. The long‐term efficacy of local excision for early colorectal adenocarcinoma in young patients is not clear. Thus, this study aimed to compare long‐term oncological outcomes of local excision with radical resection for T1 adenocarcinoma located in colon and rectum.

## Materials and Methods

The SEER Program collects and publishes data related with cancer incidence and survival outcome from population‐based cancer registries covering 28% of the US population 11. We performed a retrospective study of patients pathologically diagnosed with colon or rectal primary submucosal invasive carcinoma from 1998 to 2014 using the data from the SEER database. This study was approved by the ethics committee of our institution.

The following inclusive criteria were applied to identify those with colon or rectal cancer undergoing local excision or radical resection:(1) Patients were diagnosed from 1998 to 2014; (2) age of diagnosis was limited from 18 to 45 years old; (3) tumor site: colon cancer (C180‐C189) and rectum cancer (C199, C209) according to Third Edition of International Classification of Diseases for Oncology (ICD‐O‐3); (4) stage was based upon the American Joint Committee on Cancer (AJCC) 7th edition. Patients diagnosed with stage I (T1 [tumor invading submucosa], N0 [no regional lymph node metastasis], and M0 [no distant metastasis]); (5) histologically confirmed adenocarcinoma; and (6) underwent primary tumor resection with pathological specimen: local excision (surgical excision or endoscopic excision) or major resection (partial, subtotal, or total colectomy; anterior resection; Hartmann operation; rectosigmoidectomy; and total proctectomy). Only patients with complete results for all variables were included. The patients who received preoperative chemoradiotherapy were excluded in this study.

### Statistical analysis

The National Cancer Institute's SEER*Stat software (Surveillance Research Program, National Cancer Institute SEER*Stat software (8.3.4 version))was used to obtain all data from SEER database. All analysis was performed by R statistical software (version 3.3.3). Two‐sided *P* ≤ 0.050 was considered statistically significant. Demographic differences between two groups were tested using the Pearson chi‐square test.

To balance the potential baseline confounding variables, a propensity score matching method regarding age at diagnosis, year of diagnosis, race, gender, tumor grade, and tumor location was performed by the ““MatchIt” R package and the “nearest neighbor matching” method (ratio = 1:2). Patients underwent radical resection who did not have a counterpart regarding the distance measure among the patients underwent local excision were excluded from this study.

Kaplan–Meier method was used to plot survival curves and to calculate the CSS and OS rate. Multivariable Cox proportional hazard model was used to identified variables associated with CSS and OS. Hazard ratios (HRs) were presented with 95 percent confidence intervals (95% CI). The proportional hazards assumption for the Cox models was tested before including risk variables into Cox proportional hazards regression model. The variables which did not follow the proportional hazards assumption would be to divide into strata.

## Results

### Adjusting for patient characteristics with propensity score matching method

Total of 1292 patients with colon adenocarcinoma and 884 patients with rectal adenocarcinoma met the inclusive criteria were included in this study. Patient characteristics were showed in Table 1. Initially, significant differences were observed between local excision group and radical resection group regarding race and tumor location. The proportion of the white race in local excision group was lower than that in radical resection group (74.69% vs. 79.16%), while others’ race was larger (14.32% vs. 9.11%, *P* = 0.002). In local excision group, 45.38% of patients suffered colon cancer, significantly lower than the counterpart in radical resection group (64.38%, *P* < 0.001).

**Table 1:** Characteristics of patients included in the study

Variables	Before matching (*n *= 2176)		*P*	After matching (*n *= 1719)		*P*
	Local excision *n* (%)	Radical resection *n* (%)		Local excision *n* (%)	Radical resection *n* (%)	
Age at diagnosis						
18–40	247 (43.11)	715 (44.60)	0.568	247 (43.11)	494 (43.11)	1.000
41–45	326 (56.89)	888 (55.40)		326 (56.89)	652 (56.89)	
Year of diagnosis						
1998–2004	199 (34.73)	557 (34.75)	0.943	199 (34.73)	413 (36.04)	0.808
2005–2009	193 (33.68)	529 (33.00)		193 (33.68)	387 (33.77)	
2010–2014	181 (31.59)	517 (32.25)		181 (31.59)	346 (30.19)	
Race						
White	428 (74.69)	1269 (79.16)	0.002^a^	428 (74.69)	875 (76.35)	0.653
Black	63 (10.99)	188 (11.73)		63 (10.99)	125 (10.91)	
Others	82 (14.32)	146 (9.11)		82 (14.32)	146 (12.74)	
Gender						
Female	279 (48.69)	848 (52.90)	0.093	279 (48.69)	536 (46.77)	0.484
Male	294 (51.31)	755 (47.10)		294 (51.31)	610 (53.23)	
Tumor grade						
Well/Moderate	527 (91.97)	1456 (90.83)	0.459	527 (91.97)	1058 (92.32)	0.874
Poor/ Anaplastic	46 (8.03)	147 (9.17)		46 (8.03)	88 (7.68)	
Tumor location						
Colon	260 (45.38)	1032 (64.38)	<0.001^a^	260 (45.38)	575 (50.17)	0.068
Rectum	313 (54.62)	571 (35.62)		313 (54.62)	571 (49.83)	

a Significant *P* value.

**Figure 1:**
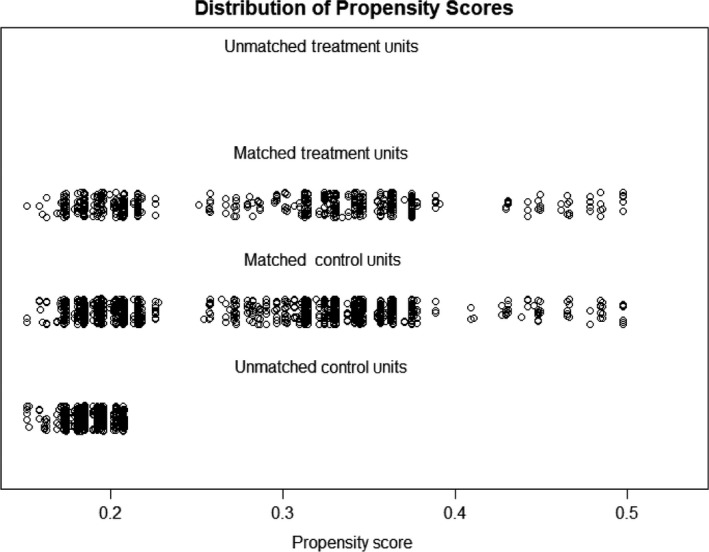
Distribution of the propensity scores. The treatment units represent local excision group, and the control units represent radical resection group. Each circle represents one patient. The size of the circles for matched patients is proportional to the distance obtained by the propensity score matching procedure. The propensity scores for patients who could not be matched with patients from the other group due to their characteristics are also shown.

After performing the propensity score matching procedure, a total of 1719 cases included in the following analysis (Fig. 1). There was no significant difference between the two groups. The median follow‐up was 80 months (IQR: 37–132), with 1074 patients followed for ≥5 years and 508 patients followed for ≥10 years.

### Kaplan–Meier and univariable cox survival analysis

**Figure 2:**
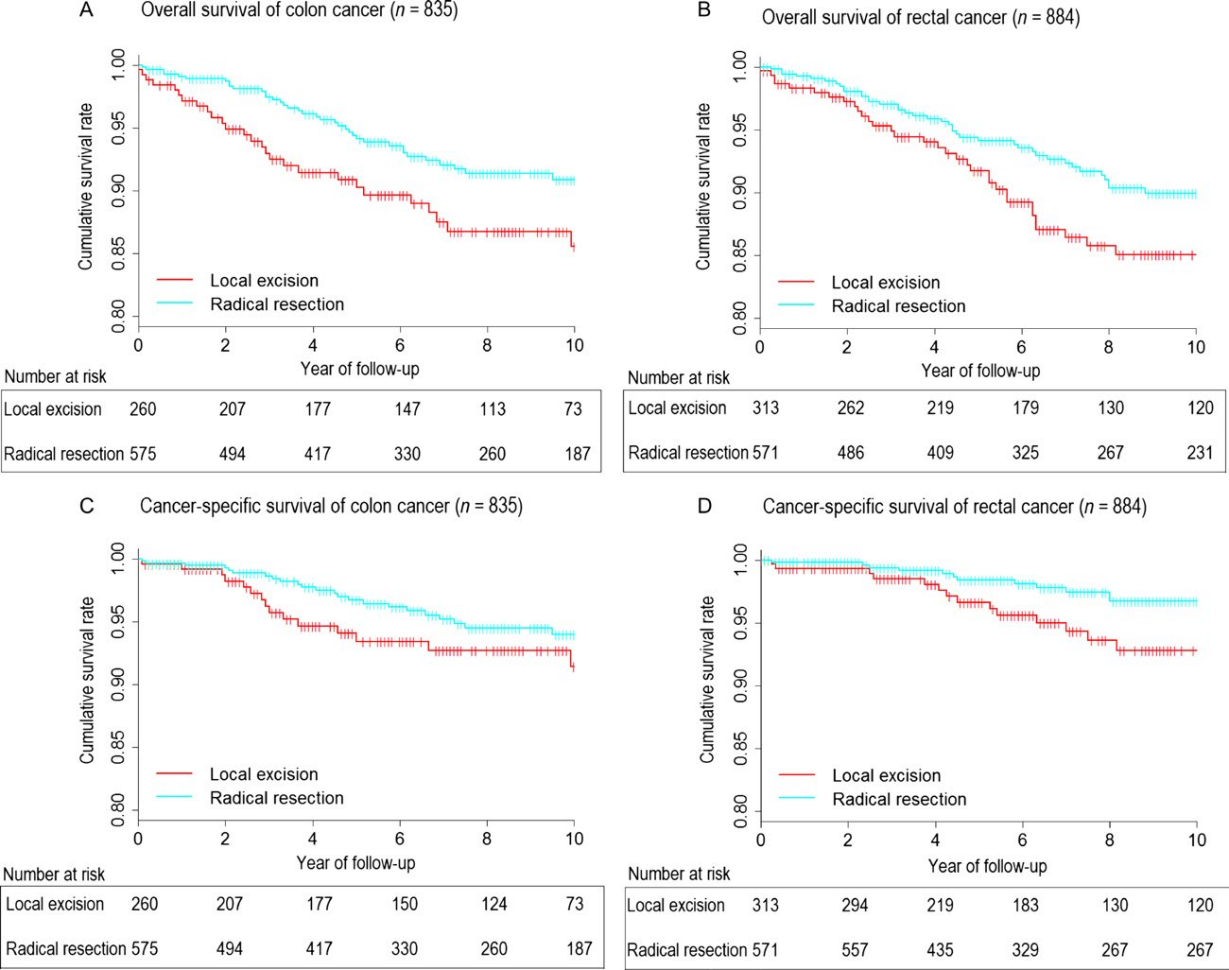
OS of local excision versus radical resection for colon cancer (A) and rectal cancer (B); CSS of local excision versus radical resection for colon cancer (C) and rectal cancer (D).

Kaplan–Meier curves displayed CSS and OS rate for colon and rectal cancer in patients underwent local excision and radical resection (Fig. 2). Five‐year CSS of local excision versus radical resection was 93.4% versus 96.7% for colon cancer and 96.6% versus 98.4% for rectal cancer. Ten‐year CSS of local excision versus radical resection was 91.4% versus 94.0% for colon cancer and 92.8% versus 96.7% for rectal cancer. Meanwhile, 5‐year OS of local excision versus radical resection was 90.3% versus 94.1% for colon cancer and 91.7% versus 94.1% for rectal cancer. Ten‐year OS of local excision versus radical resection was 85.6% versus 90.9% for colon cancer and 85.0% versus 89.9% for rectal cancer.

**Figure 3:**
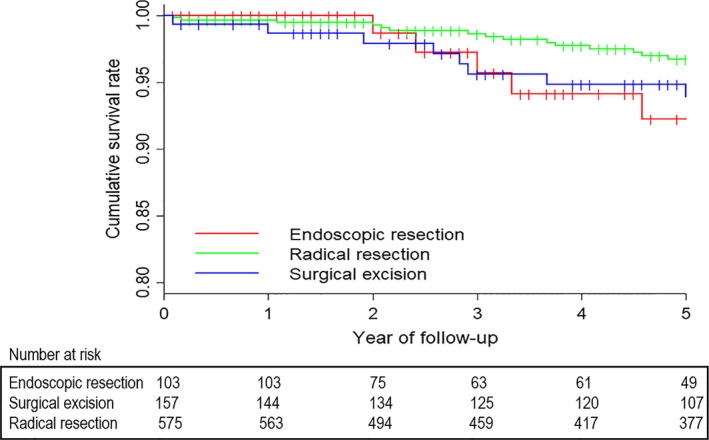
Comparison of CSS among endoscopic resection, surgical excision, and radical resection for T1 colon cancer.

The local excision group of T1 colon cancer was further divided into endoscopic resection group and surgical excision group. Then an analysis was made to compare 5‐year cancer‐specific survival outcome among endoscopic resection, surgical excision, and radical resection for T1 colon cancer (Fig. 3). Five‐year CSS was 92.2% for endoscopic resection, 93.9% for surgical excision, and 96.7% for radical resection, respectively. There was no significant survival difference was found among the three groups (*P* = 0.329).

Univariate Cox proportional hazards regression analysis demonstrated that, for colon cancer, the overall mortality risk for patients underwent local excision was increased by 70% (HR  = 1.70, 95% CI:1.05–2.73, *P* = 0.030), but no significant difference of CSS was found (HR = 1.59,95% CI:0.85–3.00, *P* = 0.149). Regarding rectum cancer, there was no significant difference of CSS (HR = 2.20,95% CI: 0.96–4.65, *P* = 0.067) and OS (HR = 1.45,95% CI: 0.92–2.28, *P* = 0.105) between local excision and radical resection. (Table 2)

**Table 2:** CSS and OS for T1 colon and rectal cancer separately

Tumor location	Survival type	Local excision (95% CI)	Radical resection (95% CI)	Unadjusted HR (95% CI)	*P* value
Colon	5‐year CSS	93.4% (90.0–97.0)	96.7% (95.1–98.4)	1.59 (0.85–3.00)	0.149
	10‐year CSS	91.4% (87.1–96.0)	94.0% (91.5–96.5)		
	5‐year OS	90.3% (86.4–94.4)	94.1% (92.0–96.3)	1.70 (1.05‐2.73)	0.030^a^
	10‐year OS	85.6% (80.4–91.1)	90.9% (88.0–93.8)		
Rectum	5‐year CSS	96.6% (94.4–99.0)	98.4% (97.2–99.6)	2.20 (0.96–4.65)	0.067
	10‐year CSS	92.8% (89.1–96.7)	96.7% (94.9–98.6)		
	5‐year OS	91.7% (88.4–95.2)	94.1% (92.0–96.3)	1.45 (0.92–2.28)	0.105
	10‐year OS	85.0% (80.3–90.0)	89.9% (87.0–93.0)		

HR, hazard ratios; 95% CI, 95% confidence intervals.

a Significant *P* value.

### Multivariable cox survival analysis

Multivariate analysis suggested that, for colon cancer, local excision was associated with increased overall mortality risk by 77% (HR 1.77, 95% CI:1.10–2.85, *P* = 0.020), but equivalent CSS (HR 1.74, 95% CI: 0.92–3.29, *P* = 0.090) (Table 3). On the contrary, local excision achieved similar OS (HR 1.40, 95% CI: 0.89–2.20, *P* = 0.148) and CSS (HR 2.16, 95% CI: 0.99–4.71, *P* = 0.052) for rectal cancer (Table 4).

**Table 3:** Multivariate analysis of OS and CSS for T1 colon cancer

Variables	Multivariate analysis of OS		*P*_test_	Multivariate analysis of CSS		*P*_test_
	HR (95% CI)	*P*		HR (95% CI)	*P*	
Age at diagnosis (year)						
18–40	Reference		0.456	Reference		0.146
41–45	0.85 (0.53–1.38)	0.517		1.13 (0.59–2.17)	0.719	
Year of diagnosis						
1998–2004	Reference		0.645	Reference		0.578
2005–2009	1.09 (0.64–1.85)	0.763		1.32 (0.65–2.67)	0.442	
2010–2014	0.61 (0.23–1.65)	0.331		0.77 (0.21–2.83)	0.697	
Race						
White	Reference		0.574	Reference		0.749
Black	2.02 (1.13–3.62)	0.018^a^		1.87 (0.85–4.12)	0.120	
Others	0.53 (0.21–1.33)	0.177		0.55 (0.17–1.81)	0.326	
Gender						
Female	Reference		0.854	Reference		0.121
Male	1.88 (1.12–3.15)	0.016^a^		1.60 (0.82–3.11)	0.171	
Tumor grade^b^						
Well/Moderate	Reference		0.542	–		0.008^a^
Poor/undifferentiation	2.00 (0.99–4.04)	0.054		–	–	
Surgery type						
Radical resection	Reference		0.237	Reference		0.368
Local excision	1.77 (1.10–2.85)	0.020^a^		1.74 (0.92–3.29)	0.090	

HR, hazard ratios; 95% CI, 95% confidence intervals; *P*_test_, test of proportional hazards assumption.

a Significant *P* value.

b Tumor grade was divided into strata in multivariate analysis.

**Table 4:** Multivariate analysis of OS and CSS for T1 rectal cancer

Variables	Multivariate analysis of OS		*P*_test_	Multivariate analysis of CSS		*P*_test_
	HR (95% CI)	*P*		HR (95% CI)	*P*	
Age at diagnosis (year)						
18–40	Reference		0.749	Reference		0.673
41–45	1.31 (0.83–2.09)	0.250		1.26 (0.57–2.80)	0.573	
Year of diagnosis						
1998–2004	Reference		0.486	Reference		0.715
2005–2009	1.20 (0.72–2.00)	0.482		1.36 (0.58–3.19)	0.480	
2010–2014	1.97 (0.94–4.09)	0.071		2.80 (0.76–10.20)	0.121	
Race						
White	Reference		0.488	Reference		0.569
Black	1.59 (0.83–3.04)	0.159		1.22 (0.36–4.14)	0.745	
Others	1.00 (0.48–2.10)	1.000		1.06 (0.31–3.61)	0.921	
Gender						
Female	Reference		0.111	Reference		0.584
Male	1.59 (1.00–2.51)	0.048^a^		1.86 (0.83–4.18)	0.133	
Tumor grade						
Well/Moderate	Reference		0.734	Reference		0.388
Poor/undifferentiation	0.90 (0.39–2.07)	0.802		1.85 (0.63–5.41)	0.264	
Surgery type						
Radical resection	Reference		0.243	Reference		0.973
Local excision	1.40 (0.89–2.20)	0.148		2.16 (0.99–4.71)	0.052	

HR, hazard ratios; 95% CI, 95% confidence intervals; *P*_test_, test of proportional hazards assumption.

a Significant *P* value.

## Discussion

In recent decades, incidence and mortality of CRC in young patients have been increasing. Although local excision for early localized CRC has been widely accepted, the long‐term efficacy of local excision for the early localized disease in the young is unclear. To the best of our knowledge, this is the first known study to investigated long‐term oncological outcomes of local excision versus radical resection for young patients with T1 CRC. SEER database offered enough data of young patients, and to be specific, total of 1719 patients, which ensured adequate power for our findings.

The main findings suggested that, compared with radical resection, 5‐ and 10‐year CSS in local excision group were similar in colon and rectal cancer, which corresponds to nonsignificant adjusted HRs by multivariable survival modeling, respectively. Hence, there was no evidence suggested that cancer‐specific mortality in local excision group was significantly higher. Although 5‐ and 10‐year CSS in local excision were slightly lower, such small difference may come at the price of huge physical wound and low living quality. These findings supported clinical application of local excision for T1 colon and rectal cancer in young adults.

As the development of endoscopic technique, endoscopic resection has become a more popular treatment for early gastrointestinal cancer, especially in China, Japan, and Korea. Survival outcomes among endoscopic resection, surgical excision, and radical resection for early colorectal cancer in young adults are unclear. In this study, 5‐year cancer‐specific survival outcome among endoscopic resection, surgical excision, and radical resection for T1 colon cancer in young adults was further analyzed. We concluded that there was no significant survival difference was found among the three groups (*P* = 0.329), which further proved that endoscopic resection could not only provided pathological information of most T1 cancer, but also be an effective treatment approach.

Whether adverse pathological features play a role in long‐term survival in patients with CRC are controversial. Brunner et al. 12 reported that poor differentiation or undifferentiation was independent risk factors for lymph node metastasis and may not be suitable for local excision. However, a large retrospective cohort study investigated that unfavorable pathological differentiation did not increase the risk of cancer‐specific mortality in patients with CRC 13. In this study, when the key baseline confounding variables were balanced, multivariate analysis suggested that poorly differentiated tumor and undifferentiated tumor were not prognostic risk factors for T1 colon and rectal cancer. In other words, unfavorable pathological differentiation may not be a contraindication for local excision. Further randomized trials are awaited.

Surgeons always catch in a dilemma when considering local excision for early colorectal cancer, especially considering endoscopic resection. A major criticism is that, compared to radical resection, positive circumferential margin rate is higher after local excision. Technological advances have contributed to an increased en bloc resection rate. It is reported that endoscopic technique achieved 91% en bloc resection rate for early CRC 14. With the popularization of endoscopic technique, en bloc resection rate can be improved in future.

Another criticism is that lymph node metastasis is difficult to be pathologically evaluated. The risk for regional lymph node metastasis is reported up to 14 percents of patients with T1 colorectal cancer 15, 16. Combing risk factors of lymph node metastasis, including large tumor size, old age, lymphatic invasion, submucosal invasion (≥1 mm), and poor differentiation, with high‐resonance magnetic imaging and computed tomography 12, 17, 18, 19, surgeons can identify patients with lower risk for lymph node metastasis, which are, by definition, clinic N0.

Hazard et al. 20 and Bhangu et al. 21 conducted two retrospective studies focusing on local excision versus radical resection for colorectal cancer in all ages based on SEER database. The former one suggested that local excision was associated with inferior CSS of T1 rectal cancer compared with radical resection. The latter one found local excision was oncologically equivalent to radical resection for T1 rectal cancer, but inferior for T1 colon. Unfortunately, these aforementioned studies were nonrandomized, and confounding variables were not balanced, which would weaken the results and conclusions to some extent. Moreover, physical functions and survival desire may be totally different between young and elder patients. The survival outcomes of all ages cannot answer whether local excision is oncologically equivalent to radical resection in young patients.

Randomized controlled trials (RCTs) are the ideal method for clinical research. Nevertheless, RCTs are restricted in major part of clinical trials due to the ethical, capital, and manpower limitations. Accordingly, cohort study becomes an important supplement in the research fields in which RCTs cannot be performed. One of the major limitations of retrospective cohort study was the unbalanced baseline confounding variables, which may lead to an unreliable result and conclusion. Propensity score matching is quite an important method to balance baseline confounders and help drawing causal inferences in retrospective study 22. To some extent, propensity score matching can make the “nonrandomized” to be “randomized” because of its ability to balance several known baseline confounders.

The major strengths of this study are as follows: (1) This was the first study focusing on long‐term oncological outcomes of local excision versus radical resection for early localized in young patients with CRC; (2) propensity score matching and multivariable analysis were performed to adjust for key confounders. Reliable results and conclusions were drawn due to the key baseline confounders were well balanced; (3) this study was population‐based and included a large number of cases, which may represent the “real‐world” outcomes. However, it also has several potential limitations: (1) Local recurrence of early colorectal cancer would be an ideal primary endpoint, but the SEER does not include record of recurrence data. Therefore, CSS and OS took the place of recurrence rate in this study; (2) positive circumferential margin after local excision always requires salvage radical resection. This part of patients belongs to neither local excision group nor radical resection group and should be excluded from this study. However, SEER database has no detailed information associated with salvage surgery; (3) lacking detail code of operation technique, further analysis of local excision by specific technique was not performed in this study. (4) Depth of submucosal invasion, lymphovascular invasion, and tumor budding were associated with lymph node metastasis, which may affect survival outcomes. However, those factors were not recorded in SEER database. Although we had balanced some key confounders, potential biases may still exist in this study.

## Conclusions

Our results provide the first evidence that local excision was oncologically equivalent to radical resection for T1 colon and rectal cancer in young patients. In the absence of RCTs, this is the best evidence to guide clinical practice of local excision for early localized colorectal cancer in young patients.

## Conflict of Interest

Bin Cao, Li Min, Shengtao Zhu, Haiyun Shi, and Zhang Shutian have no conflict of interests or financial ties to disclose.
